# Biofilm regulation in *Clostridioides difficile*: Novel systems linked to hypervirulence

**DOI:** 10.1371/journal.ppat.1009817

**Published:** 2021-09-09

**Authors:** Megan G. Taggart, William J. Snelling, Patrick J. Naughton, Roberto M. La Ragione, James S. G. Dooley, Nigel G. Ternan

**Affiliations:** 1 Nutrition Innovation Centre for Food and Health (NICHE), School of Biomedical Sciences, Ulster University, Coleraine, Co. Londonderry, Northern Ireland; 2 Department of Pathology and Infectious Diseases, School of Veterinary Medicine, University of Surrey, Guildford, Surrey, United Kingdom; University of Queensland, AUSTRALIA

## Abstract

*Clostridiodes difficile* (*C*. *difficile*) was ranked an “urgent threat” by the Centers for Disease Control and Prevention (CDC) in 2019. *C*. *difficile* infection (CDI) is the most common healthcare-associated infection (HAI) in the United States of America as well as the leading cause of antibiotic-associated gastrointestinal disease. *C*. *difficile* is a gram-positive, rod-shaped, spore-forming, anaerobic bacterium that causes infection of the epithelial lining of the gut. CDI occurs most commonly after disruption of the human gut microflora following the prolonged use of broad-spectrum antibiotics. However, the recurrent nature of this disease has led to the hypothesis that biofilm formation may play a role in its pathogenesis. Biofilms are sessile communities of bacteria protected from extracellular stresses by a matrix of self-produced proteins, polysaccharides, and extracellular DNA. Biofilm regulation in *C*. *difficile* is still incompletely understood, and its role in disease recurrence has yet to be fully elucidated. However, many factors have been found to influence biofilm formation in *C*. *difficile*, including motility, adhesion, and hydrophobicity of the bacterial cells. Small changes in one of these systems can greatly influence biofilm formation. Therefore, the biofilm regulatory system would need to coordinate all these systems to create optimal biofilm-forming physiology under appropriate environmental conditions. The coordination of these systems is complex and multifactorial, and any analysis must take into consideration the influences of the stress response, quorum sensing (QS), and gene regulation by second messenger molecule cyclic diguanosine monophosphate (c-di-GMP). However, the differences in biofilm-forming ability between *C*. *difficile* strains such as 630 and the “hypervirulent” strain, R20291, make it difficult to assign a “one size fits all” mechanism to biofilm regulation in *C*. *difficile*. This review seeks to consolidate published data regarding the regulation of *C*. *difficile* biofilms in order to identify gaps in knowledge and propose directions for future study.

## Author summary

*Clostridioides difficile* (*C*. *difficile*) is an opportunistic bacterial pathogen that causes infection of the human gut epithelium following disruption of the normal gut microflora, usually by broad-spectrum antibiotics. *C*. *difficile* infection (CDI) is recurrent in 20% to 30% of cases and can lead to significant health-related complications such as pseudomembranous colitis and, in severe cases, death. The impact and cost of this pathogen on healthcare systems are significant, and some aspects of the pathogen’s lifestyle in the host are, as yet, unknown. It is hypothesised that *C*. *difficile* exists in the gut as a biofilm due to the infection’s severity and recurrent nature. The biofilm mode of bacterial growth can protect the cells from external factors such as antibiotic treatment, physiological processes, and the immune system. However, biofilm regulation in *C*. *difficile* is not yet fully characterised, and in this review, we consolidate published primary research on *C*. *difficile* biofilm regulation to gain a comprehensive overview of the factors involved and how they may interact to enable biofilm development within a host.

## Introduction

*Clostridioides difficile* (*C*. *difficile*) is a gram-positive, rod-shaped, obligatory anaerobic bacterium with a low genome GC content (630 GC = 29.06%) [1]. This spore-forming anaerobe is found asymptomatically in 1% to 3% of healthy adult humans [2]. Additionally, it is also found in many other animals including diverse mammals, reptiles, and birds, all with the potential for zoonotic transfer to humans (for further consideration of this aspect, readers are referred to the following recent reviews [3–5]). Broad-spectrum antibiotic treatment can disrupt the normal microbiota in the gut causing dysbiosis of the gut microbiome [6,7]. This presents an opportunity for the rapid growth of *C*. *difficile*, leading to *C*. *difficile* infection (CDI) [2].

*C*. *difficile* has been ranked an “urgent threat” by the Centers for Disease Control and Prevention (CDC) in 2019 as it is the most common cause of healthcare-associated infection (HAI) in the United States of America [8,9] as well as the leading cause of antibiotic-associated gastrointestinal disease [9,10]. However, CDI has become increasingly prevalent as a community associated infection in younger, healthier patients [11,12]. Recent data on the impact of *C*. *difficile* in Europe and, specifically, the United Kingdom, are limited and have been described as urgently needed [13]. An epidemiological study on HAIs conducted between 2016 to 2017 found almost 45% of healthcare-associated gastrointestinal infections in Europe were caused by *C*. *difficile* [14].

The recurrent nature of CDI, affecting 20% to 30% of cases, adds to the already significant health service costs associated with treatment of this infection [15–17]. The real global impact of CDI is unclear; however, considering the US reported half a million CDI cases and 29,000 deaths in 2012, with an average annual cost between US$5.4 and US$6.3 billion [18], it is estimated to be significantly higher than this (for a comprehensive review, please see [19]).

**Fig 1:**
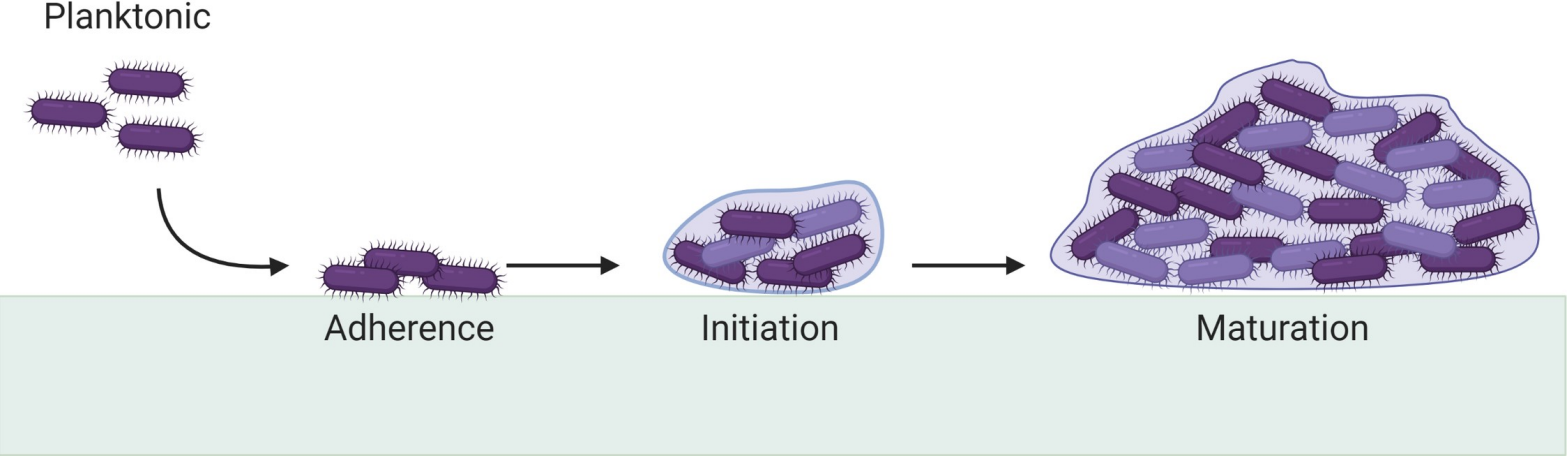
Stages of biofilm formation. Free-floating planktonic cells adhere to a surface and begin microcolony formation with production of a hydrated extracellular matrix of proteins, polysaccharides, and nucleic acids. A mature biofilm may contain subpopulations of cells existing in various states where dispersion of the biofilm may occur due to physical disruption or cell signalling. Adapted from [29]. Created with BioRender.com.

It has been hypothesised that *C*. *difficile’s* virulence and recurrence are due to its ability to form biofilms in the gut [20]. Biofilms (Fig 1) are sessile communities of microbes living within self-produced, hydrated extracellular matrices generally composed of proteins, polysaccharides, and nucleic acids [21–24]. The specific structural and compositional properties of a biofilm depend on the bacterial species, which produces the biofilm, and the environmental conditions it is exposed to [25,26]. Infections where bacteria form biofilms are characterised as persistent, chronic, and extremely difficult to treat due to the biofilms’ inherent increase in antibiotic resistance [27,28].

*C*. *difficile* has been successfully grown in multispecies biofilms in vivo with species representative of the human gut microbiome [30]. The in vitro human gut model found *C*. *difficile* spores could be conserved within the multispecies biofilms to cause recurrence [30]. In vivo studies using animal models have also shown that *C*. *difficile* is capable of forming multicellular structures such as microbial aggregates in the gut [31–34].

Further indication that *C*. *difficile* biofilms are important in pathogenesis of disease is the observation that bile salts such as deoxycholate (DOC), in the presence of fermentable sugars found in the gut, can significantly increase biofilm formation as a mechanism of the stress response [35]. Several factors influence bacterial ability to form biofilms, including adherence and motility [36–39]. Development of mutant strains of *C*. *difficile* and their use as models to assess the resulting effects of the mutations on biofilm formation have led to some understanding of how biofilms are regulated in *C*. *difficile* [20,40–44]. The aim of this review, therefore, is to consolidate and critically analyse published information on the factors known to regulate biofilm formation. We intend to provide the scientific community with a comprehensive understanding of what coordinates the necessary cellular features within *C*. *difficile* to create optimal conditions for biofilm formation.

### Cells in a sessile state

Cells within biofilms are described as being in a nonmotile, sessile state where they are attached to a surface. The switch between motile and sessile lifestyles in a wide range of bacteria—including *C*. *difficile*—is controlled by second messenger molecule, cyclic diguanosine monophosphate (c-di-GMP) [39]. c-di-GMP controls gene expression by acting on riboswitches located upstream of the controlled genes [39,45–47]. Riboswitch Cdi-1-3 is activated by c-di-GMP to down-regulate the FlgB operon containing 29 flagellar genes in the switch from motile to sessile lifestyle [45].

However, flagellar expression is also controlled by phase variation in all *C*. *difficile* strains except 630, which is effectively locked in a “flagella on” state [48]. Low levels of intracellular c-di-GMP allow for transcription of the downstream inversion site named Cdi4 in strain R20291, which can change between an “on” or “off” position [48]. DNA inversion by site-specific recombination of Cdi4 located upstream of the *flgB* operon into the “flagella off” position down-regulates flagellar expression by posttranscriptionally destabilising or degrading the mRNA potentially by a *trans*-acting element, thereby inhibiting flagellar expression on the cell surface [48].

Flagella are primarily responsible for the swimming motility of *C*. *difficile* in planktonic cultures [49–51]. Investigations of gene expression in established *C*. *difficile* biofilms compared to planktonic cultures agree that flagellar genes are decreased in expression in biofilms [38,52]. Hypervirulent *C*. *difficile* strain R20291, originally isolated from an outbreak in Stoke Mandeville Hospital, UK, formed biofilms with visibly reduced expression of flagella when grown for 7 days on glass beads [38,53]. This was reflected by a significant reduction in expression of the major flagellar filament gene, *fliC*, by 2.54-fold when compared to planktonic counterparts, indicating that flagella are down-regulated in an established biofilm [38]. Expression of several other flagellar biosynthesis genes were decreased in a 72-hour 630*Δerm* biofilm including *flhA*, *flbD*, *flgE*, and *flgD*, again showing that down-regulation of flagella components are required for biofilm formation [52]. It is unsurprising that flagella would be down-regulated when entering a sessile state, such as biofilm formation, as flagellar assembly and production is an energetically expensive process [54].

In contrast, Dapa and colleagues suggested that flagella play an important role in the late stages of biofilm formation as an R20291 *fliC* ClosTron disruption mutant exhibited decreased biofilm after 5 days compared to the parent strain [36]. However, Valiente and colleagues found that their R20291 *fliC* ClosTron mutant cells were completely lacking in flagella but had formed aggregates and mature 6-day biofilms similar to the parent R20291 strain [55]. The differences in these studies could be a result of a difference in the mutations, growth conditions, or indeed be related to the vigour of washing planktonic cells from biofilms.

Further investigations into specific glycan modifications of *C*. *difficile* flagella have also been undertaken via ClosTron mutagenesis of several glycosyltransferase genes, which are responsible for type B posttranscriptional modifications of flagella in ribotype 027 strains of *C*. *difficile*, including strain R20291 [55]. Type B modifications add glycan chains to flagella containing O-linked N-acetyl hexosamine, 2 rhamnoses with or without methylation, and a novel sulfonated peptidyl amidoglycan [55]. The addition of these glycan chains did not affect flagellar assembly in cells of R20291 but appeared to significantly increase biofilm formation and aggregation [55]. This was suggested to be due to an increase in cell hydrophobicity in the mutant *C*. *difficile* R20291 that lacked this particular flagellar posttranscriptional modification [55]. Only 2 of the 5 mutants created exhibited reduced motility, yet all 5 mutants showed a similar increase in biofilm formation. Valiente and colleagues thus concluded that motility does not heavily influence biofilm formation, but hydrophobicity, arising from the presence of glycan chains on the flagella, could enhance biofilm formation through increased aggregation [55].

### Cell surface components and adherence in *C*. *difficile* biofilms

Poquet and colleagues analysed expression of genes associated with the biosynthetic pathways of certain cell surface components in biofilm and planktonic 630Δ*erm* using microarray analysis of extracted RNA. They concluded that both the cell envelope and cell wall are modelled differently in biofilms compared to planktonic cells due to an up-regulation of phospholipid metabolism, Acyl Carrier proteins and fatty acid synthesis in the biofilm model [52]. This is similar to biofilm formation processes in *Bacillus subtilis*, in which fatty acid synthesis is required to build a hydrophobic environment within the biofilm in order to hold the complex structure together [56].

Membrane-bound glycopolymers such as PSII (Polysaccharide II) teichoic acids are conserved polysaccharide antigens, expressed at the cell surface of *C*. *difficile* [52,57]. The *lcpB* gene is involved in depositing PSII teichoic acids at the cell surface, and its expression was increased by 24.93-fold in 72-hour 630Δ*erm* biofilm cells compared to planktonic cells [52]. This could be significant as a ClosTron mutation in the *lcpB* gene in strain 630Δ*erm* significantly increased biofilm formation at 24 hours (*p* < 0.05), a phenotypic effect that was partially restored by complementation of the *lcpB* gene [44]. The *lcpB* mutant exhibited elongation and thickening of the cells along with curvature, abnormalities in septa, and slower, suboptimal growth phenomena, which have been seen in other *C*. *difficile* mutants exhibiting increased biofilm formation [41,44,58]. This could indicate that, although the *lcpB* gene itself does not regulate biofilm formation, the changes to the cell surface caused by the mutation could influence biofilm formation via knock-on effects on hydrophobicity of the cell surface and resultant cellular aggregation, similar to those observed in flagellar protein (FliC) mutants [55].

**Fig 2:**
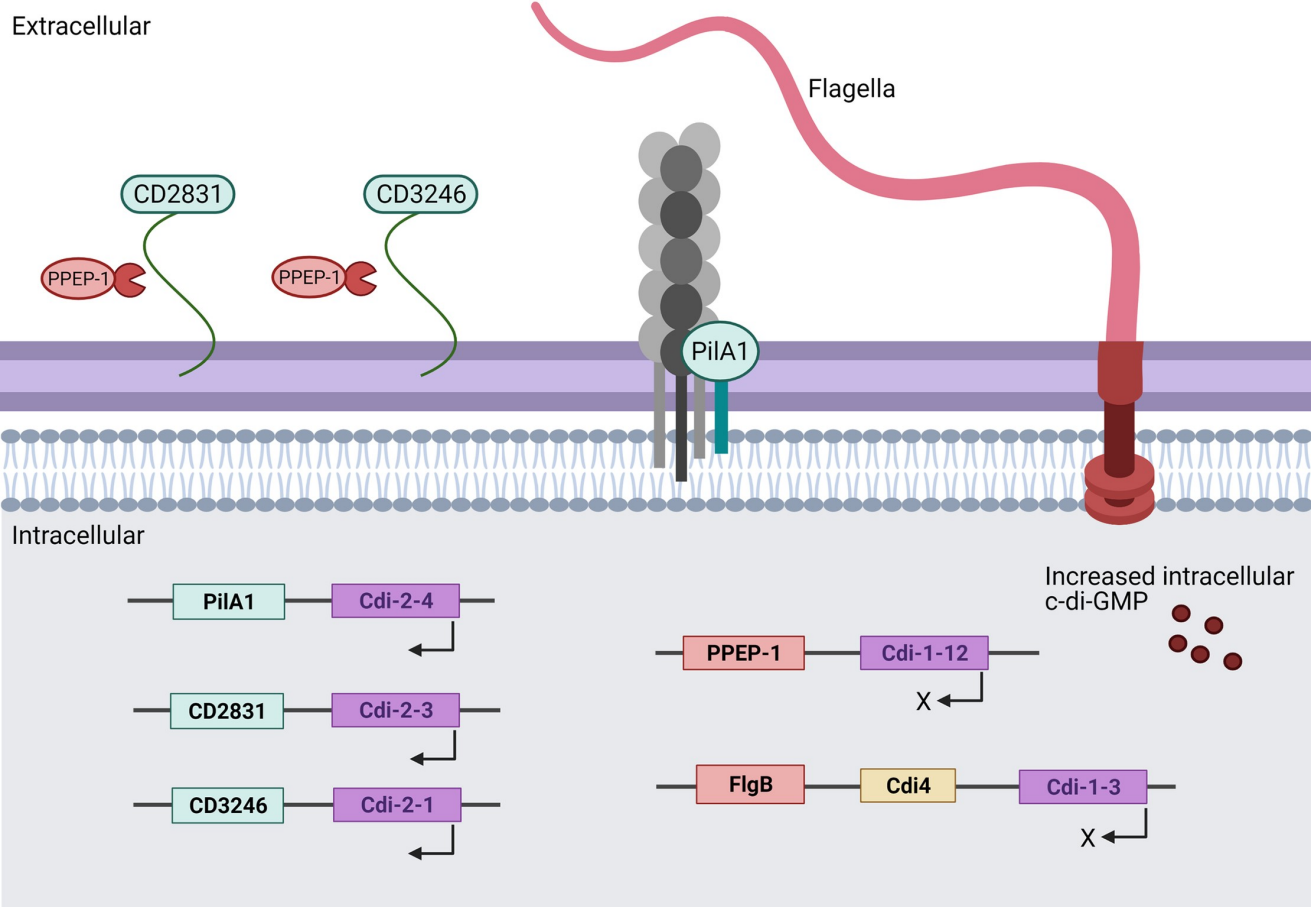
Regulation of cell surface components under high intracellular levels of c-di-GMP acting on specific riboswitches (purple). Down-regulated components in the presence of increased intracellular c-di-GMP are shown in red to include flagella and PPEP-1, which is responsible for cleavage of CD2831 and CD3246. Up-regulated components are shown in green, namely CD2831, CD3246, and PilA1, causing an increase in surface PilA1 structures. Created with BioRender.com. c-di-GMP, cyclic diguanosine monophosphate.

Other cell surface structures such as type IV pili are also known to influence biofilm formation in *C*. *difficile*, specifically with regard to their role in initial surface adherence [21,22,38,59,60]. Like the flagellar operon, the *pilA1* major pilin gene (*CD3513*) is positively regulated by c-di-GMP acting on the upstream riboswitch, Cdi-2-4 (Fig 2) [45,60]. The *pilA1* gene was up-regulated in 1-week-old biofilms along with *pilK*, *pilU*, and *pilV* when compared to planktonic growth in *C*. *difficile* R20291 [38]. A *pilA1* disruption mutant of R20291 was created that had no visible pili and that produced thinner biofilms—measured by confocal microscopy—with critically reduced biomass at a 24-hour time point [38]. However, by day 7, there was no significant difference in biofilm biomass or thickness between the *pilA1* mutant and the R20291 parent strain [38]. This led to the conclusion that pili are required for initial biofilm formation, most likely in the adhesion step [59], but not in the late stages of biofilm formation [38]. A similar effect was also seen in *P*. *aeruginosa* where, although pili-driven motility was not essential, it positively contributed to biofilm formation [61]. Further investigation of gene expression in the *pilA1* mutant at the point of adherence could help elucidate which putative adhesins are up-regulated to compensate for the lack of pili and which led to wild-type (WT) levels of biofilm formation in *C*. *difficile* R20291 at 7 days.

A separate study investigating gene expression in 630Δ*erm* biofilms at 72 hours grown in a microfermentor found that the genes for type IV pili biogenesis, including *pliA*, were up-regulated compared to planktonic cells (Table 1), in a similar pattern to that observed in 7-day-old R20291 biofilms [38,52]. However, a separate study has shown that although type IV pili affect biofilm formation in both 630Δ*erm* and R20291, they are of more critical importance in the latter as there was a higher promoter activity of the *pilA1* gene in R20291 [60]. This has led to the suggestion that the significantly increased expression of *pilA1* in R20291 biofilms when compared to planktonic culture, and which was not observed in the 630Δ*erm* model, is responsible for the increased biofilm-forming capability of R20291, which has also been reported in previous studies [36,41,60]. However, reports of 630 and 630Δ*erm* biofilms with significantly greater biofilm biomass than R20291 have also been published, which could highlight the need for standardised methods for biomass measurement [20,62].

**Table 1:** Cell surface associated component genes’ expression in 630*Δerm* 72-hour biofilm cells, compared to planktonically grown cells.

Gene ID	Gene name	Gene function/product	Fold change
**Fatty acid biogenesis**			
CD1183	*acpP*	ACP	**9.92**
CD1062	*acpP*	ACP	**9.45**
**Cell wall**			
CD2766	*lcpB*	Ligase anchoring polysaccharide to the cell wall	**24.93**
CD2762	*uppS*	Putative undecaprynyl phosphate synthetase	**−1.23**
**Exported proteins**			
CD2831		Putative adhesin	**6.77**
CD2830	*zmp1*	Extracellular zinc metalloprotease	**−1.16**
CD0873		Adhesin and sugar binding lipoprotein (ABC transport)	**65.34**
**Type IV pili biogenesis**			
CD3513	*pilA1*	Putative pilin protein	**6.11**
CD2305	*pilW*	Putative pilin protein	**11.31**
CD3504		Putative type IV prepilin peptidase, A24A family	**6.45**
CD3506		Conserved hypothetical protein	**6.19**
CD3507		Putative type IV pilin	**10.78**
CD3508		Putative type IV pilin	**12.91**
CD3509		Putative type IV pilus assembly protein	**10.13**
CD3510		Putative membrane protein	**9.78**
CD3511		Putative type IV pilus secretion protein	**9.25**
**Flagellum biogenesis**			
CD0234	*csrA*	Carbon storage regulator homolog CsrA	**−1.35**
CD0254	*flgD*	Basal-body rod modification protein FlgD	**−1.24**
CD0255	*flgE*	Flagellar hook protein FlbE (distal rod protein)	**−1.29**
CD0255A	*flbD*	Flagellar protein FlbD	**−1.28**
CD0263	*flhA*	Flagellar biosynthesis protein FlhA	**−1.19**

630Δ*erm* biofilm was grown in a continuous flow microfermentor for 72 hours with gene expression measured by microarray. Negative integers represent decreased expression. (Data from [52] transformed to show absolute fold changes).

ACP, acyl carrier protein.

Additional proteins with an adhesin function in *C*. *difficile* including *cwp66* and *CD0802* were both down-regulated in 7-day-old R20291 biofilms propagated on glass coverslips compared to planktonic cells [38]. Expression of the cell wall–binding cysteine protease, *cwp84*, wasn’t significantly different between the biofilm and planktonic grown cells of strain R20291 [38]; however, when the gene was disrupted by a ClosTron insertion in this strain, biofilm formation was dramatically decreased at 1, 3, and 5 days (*p* < 0.05) [36]. Alternatively, another study in 630Δ*erm* measured a significant increase in biofilm formation by a *cwp84* mutant (*p* < 0.05) [37]. This may indicate that *cwp84*, like *pilA1*, is regulated differently in 630Δ*erm* and R20291 [60] and therefore could play a more significant role in R20291 biofilm formation. A comparative controlled study would be required to investigate this.

The Cwp84 protease is also responsible for cleavage of SlpA into the 2 S-layer protein subunits that form the outer S-layer cover on vegetative *C*. *difficile* cells [63–65]. Richards and colleagues investigated the influence of the S-layer on biofilm formation by creating deletion mutations in the SLCT-11 S-layer cassette of *C*. *difficile* strain Ox247 (ribotype 005) [40]. Mutations in *orf2*, *orf3*, *orf4*, *orf7*, *orf16*, and *orf19* of the SLCT-11 caused an average decrease in cell length of approximately 15%, and the ability to sporulate was eliminated in *orf2* and *orf19* mutants [40]. While the *orf2* mutant was more weakly adherent to Caco-2 cells, it was the only mutant that formed significantly more biofilm in 24-well microtiter plates at 24- and 72-hour time points compared to the parent strain (*p* < 0.05) as quantified using the crystal violet assay [40]. It was concluded that the effects on biofilm formation of mutating *cwp84* in *C*. *difficile* were not due to the effect this has on SlpA [36,37,40].

Several cell surface associated proteins in *C*. *difficile* are regulated by c-di-GMP including CD2795 (cell wall binding protein, Cwp11), CD2796 (cell surface protein, Cwp10), and CD2797 (calcium-binding adhesion protein) [45]. CD2795, encoding Cwp11, has been identified as a protein released in the “secretome” during biofilm formation, while *cwp10* expression was found to be increased by 9.13-fold in 630*Δerm* biofilms when compared with planktonic cells [37,52].

Cell wall adhesin CD2831 is characterised as a microbial surface component recognising adhesive matrix molecule (MSCRAMM) as it has been found to bind to immobilised collagen on ELISA plates [39]. CD2831 and CD3246 are both cell surface proteins positively regulated by c-di-GMP through riboswitches Cdi-2-3 and Cdi-2-1, respectively [45,62]. Both are cleaved from the cell surface by CD2830, a zinc-dependent metalloprotease (Zmpl) named PPEP-1 (pro-pro endopeptidase), which is negatively regulated by c-di-GMP acting on riboswitch Cdi-1-12 [39,62,66,67]. Cdi-2-1 up-regulates CD3246, a cell surface protein that produced a significant increase in biofilm formation when expressed ectopically using an inducible plasmid [45].

CD2830 was found to be the most down-regulated gene—by 62.61-fold—when c-di-GMP levels were artificially increased using an inducible plasmid, whereas CD2831 was the most up-regulated gene (by 42.51-fold) [45]. This correlates well with observations of gene expression in 630*Δerm* biofilm compared to planktonic cells (Table 1), where *CD2831* expression increased by 6.77-fold and *CD2830* expression decreased by 1.16-fold [52].

Overexpression of CD2831 using an inducible promoter on a plasmid increased 630Δ*erm* biofilm proportionately [39]. Similarly, a ClosTron mutant of CD2831 in strain 630 showed a significant reduction in early biofilm at 24 hours (*p* < 0.05) but did not exhibit significant effects on attachment or late biofilm formation [62]. This mutation could be complimented using an inducible plasmid containing the *CD2831* gene to restore biofilm to the original phenotype [62]. Furthermore, increased expression of *CD2831* or *CD3246* using an inducible plasmid in a PPEP-1 mutant of strain 630 caused a significant increase in early biofilm formation ((*p* < 0.01) or (*p* < 0.05), respectively) [62].

Other cell surface structures, namely CD3392, CD0183, and CD3145, which are not regulated by c-di-GMP, also produced significant reductions in biofilm formation (*p* < 0.01, *p* < 0.001, and *p* < 0.05, respectively) when genetically disrupted by ClosTron mutagenesis [62]. CD3392 is a cell wall protein containing an SPKTG motif anchored to the cell wall via sortase, which was present in the biofilm matrix of strain 630Δ*erm* [62]. Whereas CD0183 and CD3145 (CbpA) are anchored to the cell wall independently of sortase but contain SPSTG and SPQTG motifs, respectively [62,68]. A role for CD0183 and CbpA in biofilm formation has yet to be determined [47,62,69]; however, CbpA is a novel cell surface exposed adhesin with a high affinity for binding to collagens I and V [62,69], and CD0183 with suspected autolytic properties has been shown to be positively controlled by small RNA RCd2, which itself is induced when cells enter exponential growth phase [47].

Taken together, the above observations by a range of independent groups suggest that CD2831 and CbpA could play a role in pathogenesis by binding to native collagen-producing cells found on the host extracellular matrix after toxin activity has exposed the fibronectin and collagen [39,69,70]. CD2831 may also aid virulence by inhibiting the formation of the C1 complement complex by binding to the C1q component of the host complement pathway [39], thereby limiting the host’s immune response. This observation is important for understanding of the pathogenesis of *C*. *difficile* infection and disease as it suggests that CD2831 and CpbA could be responsible for *C*. *difficile* adherence to the gut epithelium via host collagen exposed through the action of *C*. *difficile* toxins, while at the same time possibly limiting the immune response.

### Toxin production in *C*. *difficile* biofilms

*C*. *difficile tcdA* and *tcdB* genes encode toxin A and toxin B proteins, respectively, and are located within the pathogenicity locus (PaLoc) of the genome [71]. The other genes required for toxin synthesis are also encoded within PaLoc; *tcdR* and *tcdC* are both involved in regulation and *tcdE*, which has a role in toxin secretion [72,73]. Toxin production by *C*. *difficile* is positively controlled by up-regulation of *tcdR* expression by effect of sigma factor *sigD* (originally annotated and named by Aubry and colleagues as *fliA*) [74,75]. However, *sigD* is negatively controlled by c-di-GMP levels and is also responsible for regulation of the *fliC* gene [74]. Consequently, *sigD* has been identified as a key virulence regulator as it can lead to decreased expression of both flagella and toxin production under elevated c-di-GMP levels [74].

An RNA-seq comparison of gene expression in *C*. *difficile* R20291 biofilm and planktonic cultures revealed a statistically significant increase in *tcdB* expression by 2.83-fold (*q* < 0.05) in biofilms compared to planktonic culture, but without a corresponding increase in *tcdA* expression [38]. However, when comparing toxin expression in biofilm compared to colony growth, both *tcdA* and *tcdB* exhibited significant decreases in expression in the biofilm culture (by 3.25-fold and 2.89-fold, respectively) [38]. Differential expression of *tcdA* and *tcdB* was also found in *C*. *difficile* 630Δ*erm* biofilms grown in a continuous-flow microfermentor for 72 hours [52]. However, in this case, *tcdA* expression had decreased by only 1.03-fold, and *tcdB* expression was not significantly different [52]. The reported differences between these studies, with respect to the bacterial strains used, culture conditions, and time of RNA extraction make direct comparisons difficult. However, it raises the possibility that, under comparable conditions, the 2 strains may well behave similarly with regard to toxin gene expression in biofilms [38,42,76].

In *C*. *difficile* 630 cells cultured in human faecal water, expression of *tcdA* was decreased by 4-fold (*p* < 0.001) [77]. It was suggested that this was due to a decrease in metabolism of carbohydrates into butyrate [77]. Nonetheless, while toxin expression decreased in the presence of FW, the expression of sporulation genes significantly increased—by up to 300-fold in some cases [77]. This indicates that FW components induce sporulation, potentially aiding the transmission of *C*. *difficile*, but that FW components alone do not necessarily play a role in disease onset by inducing toxin production [77].

### Sporulation in *C*. *difficile*

*C*. *difficile* produces metabolically dormant structures called spores that are resistant to oxygen and heat stress as well as some disinfecting agents [78,79]. Spores, as the transmissible agent, are therefore important for the spread of *C*. *difficile* as they allow the pathogen to survive outside of the host [78–80]. The Spo0A master regulator in *B*. *subtilis* is responsible for driving 3 possible pathways: sporulation, production of an extracellular matrix, or production of toxins, which sacrifice some cells in order to gain nutrients, a process referred to as cannibalism [20,36,81–83]. Low levels of phosphorylated Spo0A causes matrix production in *B*. *subtilis*, whereas increasing phosphorylation results in spore production [81]. Disruption of *spo0A* in *C*. *difficile* strain R20291 resulted in complete inhibition of sporulation and significantly smaller biofilms than the parent strain at 3 and 6 days (*p* < 0.05) when grown in microtiter plates as measured by crystal violet assay [20]. These smaller *spo0A* mutant biofilms were more easily detached and dispersed when gently agitated in tissue culture flasks, compared to the parent strain biofilm [20]. This reduction in biofilm in the *C*. *difficile* R20291 Spo0A mutant led the authors to conclude that the role of *spo0A* in *C*. *difficile* is analogous to that of the Spo0A regulator in *B*. *subtilis* [20]. However, the effects on biofilm formation and dispersal could also be an indication that the *spo0A* regulator mutation influences other processes such as adherence, an aspect not examined by Dawson and colleagues in their 2012 paper [20].

A more recent study by Dawson and colleagues examined sporulation frequency within biofilms for 5 *C*. *difficile* strains including 630 and R20291 and found similarities in biofilm composition between strains [62]. A positive correlation between sporulation frequency and biofilm biomass was found along with a positive correlation with eDNA in the matrix [62]. It was suggested that the increased eDNA and sporulation correlating with increased biofilm biomass could arise from induction of sporulation within the biofilms resulting in cell lysis to release eDNA and intracellular proteins into the matrix [62]. The importance of eDNA as a key component in *C*. *difficile* biofilms was proven by successful inhibition and disruption of *C*. *difficile* biofilms using DNase enzymes [35,62]. However, identification of other proteins in the matrix, namely Cwp19 and phage protein (phiCD24-1), which both contribute to cell lysis and so would also elevate eDNA, indicates a complexity to the biofilm formation process and can explain why disruption of single genes cannot produce an all-or-nothing biofilm phenotype where many processes are involved [62]. Additionally, the authors also found that 630 and R20291 had significantly greater numbers of spores in their biofilms, compared to planktonic supernatants, which were suggested to aid recurrence of infection through dispersal of the biofilm and reseeding spores elsewhere in the gut [62].

A study performed by Tijerina-Rodríguez and colleagues to determine the differences in recurrent-CDI (R-CDI) cases and nonrecurrent-CDI (NR-CDI) clinical samples found that there was no significant difference in distribution of *C*. *difficile* ribotypes between R-CDI and NR-CDI samples; however, 81% of 102 samples examined contained RT-027 strains of *C*. *difficile* [84]. Significantly higher rates of sporulation were found in R-CDI isolates compared to NR-CDI isolates grown in 7-day mono-biofilms (*p* = 0.015), which corresponded with R-CDI biofilms having higher expression of both *spo0A* and *sigH*, the alternative sigma factor for controlling sporulation [84]. This also fits with the theory of Dawson and colleagues discussed above that biofilms with increased sporulation frequency can reseed and lead to recurrent infections [62]. Crucially, however, there was no significant difference in biofilm formation between the 2 groups (R-CDI and NR-CDI) at 7 days as measured by the crystal violet assay [84]. This suggests that increased *spo0A* expression does not influence biofilm formation significantly and that sporulation may play a larger role in recurrence of disease than biofilm formation [84].

### The role of the stress response in *C*. *difficile* biofilms

Bacteria respond to stressors, such as adverse and fluctuating conditions, by altering gene expression to adapt to new environments [85]. Biofilm formation by bacteria is recognised as a stress response, as the production of an extracellular matrix shields the bacterial cells from external pressures such as oxygen stress and antibiotics [20,86–88]. Several factors within the biofilm can contribute to recalcitrance to antibiotics such as polarity of the matrix inhibiting drug diffusion and reduced metabolic activity within the cells preventing antibiotic uptake into the cells [27].

Subinhibitory antibiotic concentrations are known to cause a stress response in many bacteria including *Escherichia coli*, *Vibrio cholerae*, and *Staphylococcus aureus*, where they also lead to increased expression of virulence factors [89]. *C*. *difficile* strains that showed susceptibility to antibiotics such as metronidazole produced significantly more biofilm in the presence of subinhibitory concentrations of this antibiotic [86,90]. The regulatory processes and mechanism underpinning this observation were not confirmed in these studies; however, subinhibitory concentrations of antibiotics have been found to trigger the stress response in other bacteria [89]. Consequently, treatment of CDI with lower concentrations of ineffective antibiotics could worsen the disease state or lead to recurrence.

A pattern of stress responses inducing morphological changes is seen in many bacterial species [89]. For example, a disruption mutation in the *prkC* gene in 630Δ*erm* resulted in the mutant producing significantly increased biofilm under bile salt–induced stress conditions (*p* < 0.05) [57]. The *prkC* gene encodes a membrane-associated serine/threonine (Ser/Thr) kinase enzyme with 2 penicillin-binding and Ser/Thr kinase-associated (PASTA) domains, but its function is, as yet, uncharacterised in *C*. *difficile* [57]. The *prkC* mutant strain produced significantly increased biofilm after 24 hours, but only in the presence of compounds that stressed the cell envelope, such as the bile salt DOC, or the antibiotic polymyxin B [57]. Other effects caused by the mutation were a >100% elongation of the cells, in which abnormal septa were identified by confocal and transmission electron microscopy, along with replication defects, reduced motility, and increased aggregation [57].

The DnaK protein is also an important component of the *C*. *difficile* stress response and has also been found in the biofilm matrix [58,62]. It is a molecular chaperone that aids protein folding and is up-regulated under heat stress conditions [91]. A ClosTron insertion mutation in the *dnaK* gene of 630Δ*erm* disrupted DnaK production and generated a less thermotolerant strain that exhibited suboptimal growth at 37°C and reduced motility due most likely to the lack of flagella on the cell surface as revealed by TEM [58]. However, disruption of the *dnaK* gene also resulted in a significant increase in biofilm formation, as well as approximately 50% elongation of the cells into filamentous rods [58].

Similar effects were seen when other elements of the *C*. *difficile* stress response were investigated. The SOS response is a regulatory network within the stress response of bacterial cells that reacts to cellular damage and, in particular, DNA damage [41,85,89]. The SOS response is regulated by proteins LexA and RecA [89]. The global transcriptional repressor, LexA, temporarily halts cellular division in *C*. *difficile* and activates processes for cellular DNA repair [41,92]. Disruption of the *lexA* gene in *C*. *difficile* R20291 resulted in elongated, filamentous cells, as well as increased biofilm production compared to the parent strain [41]. These observations are similar to those made with the *dnaK* mutant in 630Δ*erm*, thus emphasising the effects of stress on biofilm formation [58].

While physiological stress is clearly implicated in biofilm formation, other signalling processes initiated by favourable growth conditions that result in an increase in cell number and density are also present in bacteria [93,94]. This method of communication is referred to as quorum sensing (QS).

### The role of quorum sensing in *C*. *difficile* biofilms

**Fig 3:**
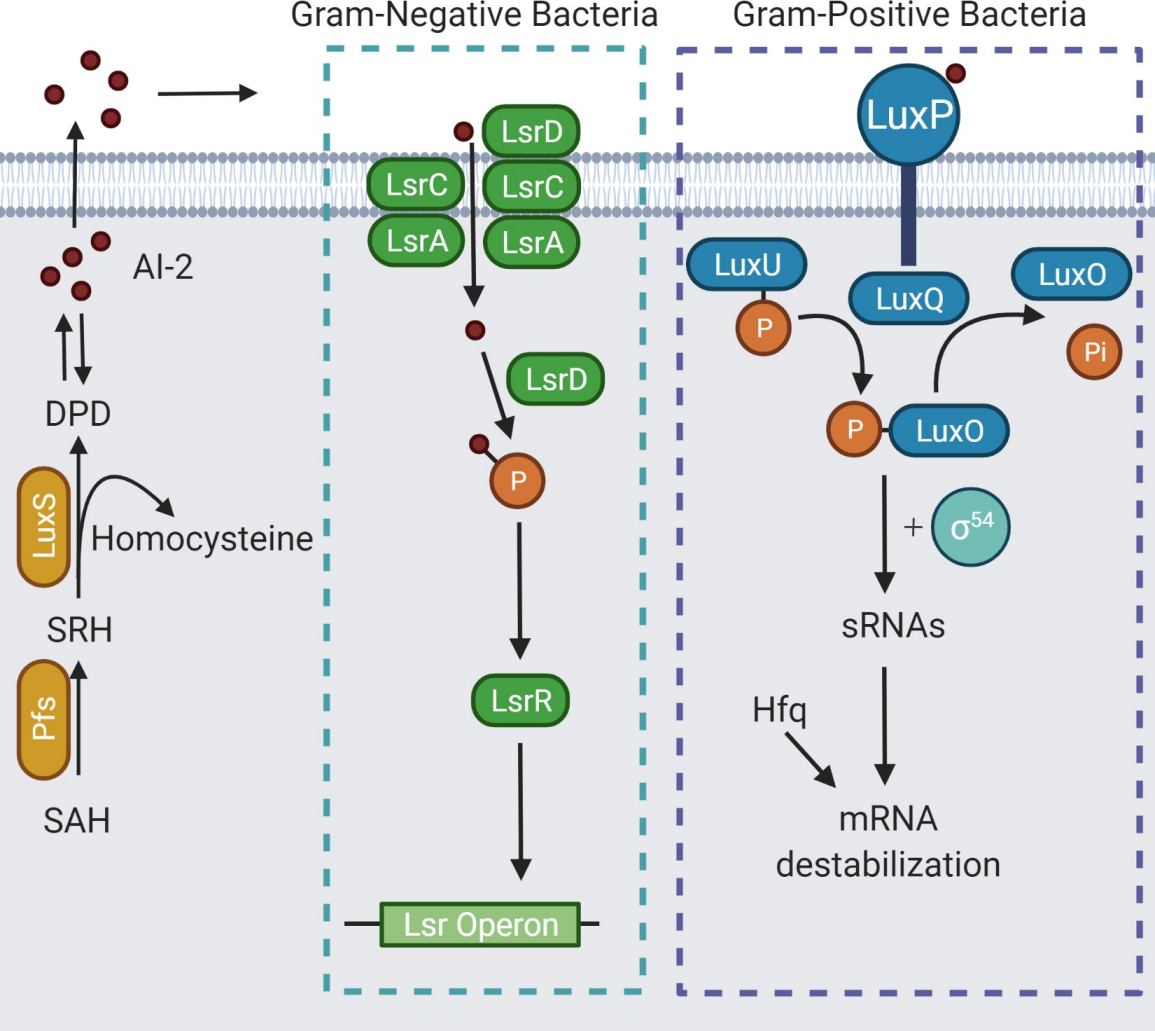
Overview of the LuxS QS system in both gram-negative and gram-positive bacteria. Both systems use the same signalling molecules, collectively named AI-2. Created with BioRender.com adapted from [96]. AI-2, autoinducer-2; QS, quorum sensing.

QS is a system that regulates gene expression in bacterial cells by using signalling molecules called autoinducers that build up as a direct result of increasing cell population density [93,94]. The LuxS QS system exists in both gram-positive and gram-negative bacteria and uses signalling molecules collectively known as autoinducer-2 (AI-2) [95,96]. AI-2 molecules act on cell surface receptors not yet identified in *C*. *difificile* to generate an intracellular signalling cascade that results in gene regulation (Fig 3) [42,95,96].

A *luxS* ClosTron mutant in the hypervirulent *C*. *difficile* strain R20291 was defective in production of AI-2 molecules, as measured by induction of bioluminescence in the bioreporter strain *Vibrio harveyi* BB170, which responds only to AI-2 signals [42,97]. As a result of this mutation in *luxS*, the mutant R20291 was also deficient in biofilm formation [42]. Chemical complementation using the compound 4,5-dihydroxy-2,3-pentanedione (DPD), which spontaneously and reversibly forms AI-2, showed that a concentration of 100 nM DPD could restore biofilm formation to WT levels, thereby confirming that both AI-2 and LuxS are involved in biofilm formation in *C*. *difficile* [42,95]. However, the receptor for AI-2 in *C*. *difficile* has yet to be identified [42]. RNA-seq analysis of gene expression in the R20291 *luxS* mutant biofilm cells revealed 3 genes with increased expression: CDR20291_2554 (*crr*), a PTS system glucose-specific transporter subunit IIA; CDR20291_2927, a cellobiose phosphate-degrading protein; and CDR20291_2930 (*treA*), a trehalose-6-phosphate hydrolase [42]. A total of 18 genes exhibited decreased expression compared to the WT; these genes corresponded, in the main, to 2 putative prophage regions [42]. The prophage regions, therefore activated by AI-2, have been linked to induction of cell lysis that produces eDNA, which, as mentioned above, is necessary for biofilm formation in *C*. *difficile* [36,42,62,98].

However, a separate study that investigated gene expression in biofilms, planktonic cells, and colony-grown *C*. *difficile* R20291 reported no significant difference in expression of the *luxS* gene between the growth models [38]. It was suggested that this could be due to the RNA being extracted from a 7-day-old biofilm in the later stages of growth, where it might be expected that there would be differences in gene expression compared to the early stages of biofilm formation [38]. AI-2 production by planktonic *C*. *difficile* has been shown to increase to a maximum at 8 hours, correlating to mid to late log phase of growth, declining thereafter [76]. Lee and Song did not, however, investigate biofilm formation alongside this observation. Biofilm initiation in *C*. *difficile* has been shown to occur between 6 and 12 hours, which could feasibly correlate with the peak in AI-2 seen at 8 hours [38,42,76,90].

In hypervirulent *C*. *difficile* strains such as those belonging to RT-017 or RT-027, the whole *agr* operon, *agrACDB* (*agr2*), encodes another QS system [53,99,100]. This is similar to the *agr* QS locus, *agrACDB* in *S*. *aureus*, which is a global regulator of virulence gene expression [99]. However, the partial locus, *agrDB* (*agr1*), has been found in all *C*. *difficile* genomes analysed to date [53,100]. The *agr2* QS system uses the small secreted cyclic autoinducing peptide (AIP) encoded by *agrD*, which is processed and exported by the transmembrane protein encoded by *agrB* [99]. A study investigating the difference between clinical isolates of *C*. *difficile* from R-CDI cases and NR-CDI cases found no significant difference in biofilm formation between the 2 groups but found a significant increase in sporulation in R-CDI isolate biofilms (*p* = 0.015) [84]. There was also no significant difference in *luxS* expression; however, *agrD1* expression was significantly increased by 14.02 relative to 16S rRNA expression (*p* = 0.001) in R-CDI isolates when compared to NR-CDI isolates [84].

The *agr* QS system has also been associated with toxin regulation as allelic exchange deletion mutants of *agrB1D1* in the *agr1* partial locus of 630 and R20291 were deficient in both toxin A and B production [100]; an *agrA* insertional inactivation (ClosTron) mutation in R20291 also resulted in significantly reduced expression of *tcdA* compared to the parent R20291 strain as well as reduced flagellar operon expression [99]. On the other hand, an R20291 *luxS* disruption mutant deficient in AI-2 production exhibited no change in toxin RNA expression compared to the WT, indicating that the LuxS QS system has perhaps minimal influence on toxin production in *C*. *difficile* [42]. This is reinforced by the differences in expression of *tcdA* and *tcdB* when *luxS* expression remains unchanged in R20291 biofilms grown for 7 days on glass beads [38].

The insertion mutation in the *agrA* gene of *C*. *difficile* strain R20291 also resulted in decreased expression of 3 genes encoding diguanylate cyclase (DGC) and phosphodiesterase (PDE) enzymes responsible for c-di-GMP production [99]. This implies that the *agr* QS system is also involved with in regulation of production of c-di-GMP [99].

### The regulation of c-di-GMP in *C*. *difficile*

Soutourina and colleagues hypothesised that biofilm formation and motility in *C*. *difficile* is controlled by c-di-GMP acting on riboswitches, a type of small noncoding RNA molecule which, in *C*. *difficile* and other bacteria, act directly or indirectly on mRNA to control protein production [39,45–47]. Upon binding of c-di-GMP to a riboswitch, transcription of the associated mRNA molecule can be decreased by premature termination or positively controlled via a complex alternative splicing mechanism [47]. Small increases of 2- to 6-fold in intracellular concentrations of c-di-GMP by a nisin-inducible plasmid in strain 630 compared to empty vector control have been shown to be sufficient to cause a change in gene expression in *C*. *difficile* as exemplified by a significant reduction in swarming motility [101]. However, it has also been reported that cellular response to c-di-GMP levels within the cell can vary between *C*. *difficile* strains, for example, R20291 and 630, as R20291 has some of the lowest number of metabolic genes controlled by c-di-GMP [101,102]. Intracellular c-di-GMP levels are controlled by the actions of DGC and PDE enzymes [45].

A nisin-inducible plasmid overexpressing *dccA*, encoding a DGC enzyme responsible for the conversion of 2 guanosine triphosphate (GTP) molecules into c-di-GMP, has been used to increase intracellular c-di-GMP levels [45,102]. Artificial fluctuations in intracellular c-di-GMP concentration have been shown to influence swimming motility and flagellar gene expression, as well as aggregation and type IV pili in 630Δ*erm* (Table 2); therefore, it is unsurprising that increasing intracellular c-di-GMP concentrations significantly increased biofilm formation at 24 hours as measured by crystal violet staining [8,45,59,60,101]. A plasmid containing *dccA* induced by anhydrotetracycline (ATc) was used to increase c-di-GMP levels in strain 630 [62]. A significant increase in early biofilm formation at 24 hours was found (*p* < 0.001 with 25 ng/ml ATc), but by 72 hours, the mature biofilms with increased c-di-GMP showed no significant increase in biofilm formation compare to the WT [62]. Therefore, it was concluded that c-di-GMP plays a greater role in early biofilm formation rather than the later stages [62]. However, other effects of artificially increasing intracellular c-di-GMP have also been reported such as defective cell separation, resulting in chains of *C*. *difficile* cells with abnormal septa, similar to the cell elongation mentioned above in *dnaK*, *lexA*, *prkC*, and *lcpB* mutant strains, which all exhibited increased biofilm formation [41,44,57,58,66].

**Table 2:** Changes in c-di-GMP riboswitch-controlled gene expression resulting from increased intracellular c-di-GMP in *Clostridioides difficile* strain 630Δ*erm*.

Class	Riboswitch	Downstream gene	Encoded protein	Fold change
Class I	Cdi-1-1	CD1990	SH3 domain containing protein	31.09
	Cdi-1-2	CD2797	Calcium binding adhesion protein	−7.14
	Cdi-1-3	CD0245	FlgB operon encoding 29 flagellar genes	−15.06
	Cdi-1-8	CD19903	Hypothetical protein	−7.78
	Cdi-1-9	CD2309	Hypothetical protein	−4.66
	Cdi-1-11	CD33682	Hypothetical protein	−8.90
	Cdi-1-12	CD2830	Zmpl/PPEP-1	−62.61
Class II	Cdi-2-1	CD3246	Cell surface protein	4.29
	Cdi-2-2	CD3267	Two-component response regulator	18.78
	Cdi-2-3	CD2831	Putative adhesin predicted to bind collagen	42.51
	Cdi-2-4	CD3513	Type IV pili (PilA1)	11.74

Fold changes in gene expression were measured by microarray analysis of planktonically grown 630Δ*erm* expressing plasmid encoded pDccA compared to a vector control. Plasmid induced expression of *dccA* by 1 μg/ml nisin was used to increase intracellular c-di-GMP levels. (Adapted from [45]).

c-di-GMP, cyclic diguanosine monophosphate; PPEP-1, pro-pro endopeptidase-1; Zmpl, zinc-dependent metalloprotease.

It is unknown if the extent of the artificial increase in intracellular c-di-GMP by the inducible plasmid is likely under physiological conditions, so what is seen could be an overestimation of the effects, whereas the observations of gene expression within a biofilm model, in which c-di-GMP was not artificially influenced, could represent a more realistic assessment of the effects of c-di-GMP [45,52]. Some studies have reported undetectable levels of c-di-GMP in strain 630 using liquid chromatography–tandem mass spectrometry (LC–MS/MS) with a limit of detection of 0.5 ng/mg dry weight [66]. Nonetheless, Purcell and colleagues recorded levels of c-di-GMP in planktonic *C*. *difficile* 630 cells to be 15 to 50 nM, which is low in comparison to other bacteria [101,103].

The effect of nutrient availability on intracellular c-di-GMP concentrations could explain why *C*. *difficile* biofilm formation is enhanced in the presence of glucose in the media [8,90]. Nutrient availability modulates CodY (CD1275), a transcriptional repressor that is activated by GTP [8]. However, GTP is also required to make c-di-GMP in a process that is regulated by PdcA, a PDE enzyme that degrades c-di-GMP [8]. Interestingly, the PdcA enzyme is also regulated by CodY but is dependent on GTP concentrations [8]. Therefore, at low GTP levels, PdcA transcription is active to produce PdcA enzyme molecules, which break down c-di-GMP into GTP. However, with high GTP levels, CodY is activated and acts to down-regulate PdcA transcription to prevent PdcA enzyme-mediated degradation of c-di-GMP in a negative feedback loop. CodY has also been found to bind to the *tcdR* toxin gene promoter in the presence of GTP, also linking increased toxin expression to elevated nutrient availability [104]. Consequently, increasing nutrient availability increases c-di-GMP levels, which can, in turn, act on relevant riboswitches to promote biofilm formation in *C*. *difficile* [8,45].

Regulation of *dccA*, a DGC that synthesises c-di-GMP and CD1412, a putative PDE that degrades c-di-GMP, has also been linked to the CD2214-2215 two-gene operon [45,47,52]. In 630, CD2214 encodes a protein homologous to the biofilm and sporulation repressor, *sinR*, from *B*. *subtilis* [52,105]. When disrupted by ClosTron mutagenesis in strain 630Δ*erm*, the CD2214-2215 two-gene operon was found to regulate many pathways including sporulation, the stress response, toxin synthesis, and membrane transport as well as metabolism and motility potentially through the effects of c-di-GMP [52,106]. Therefore, it has been suggested that the CD2214-CD2215 two-gene operon could be responsible for the regulation of some 15% of genes that are differentially expressed in biofilm formation, including genes involved in cellular metabolism and transition between growth stages [52].

## Conclusions

Biofilm formation and bacterial over growth in the gut is a serious threat because of increasing antibiotic resistance, making treatments less effective [107]. The complexity of *C*. *difficile* biofilm regulation and the differences between strains clearly indicate that there will almost certainly be no “silver bullet” solution to this growing and challenging problem. This review is timely in that it has identified stark differences in regulatory systems between the most intensely investigated *C*. *difficile* strains, namely 630 and R20291, making it difficult to assign a “one size fits all” paradigm to biofilm regulation in this important organism. Although R20291 has a lower number of metabolic genes controlled by c-di-GMP than 630 [101,102], there appears to be a regulatory link between c-di-GMP and the full *agr1* QS system that is found in R20291, which is not found in 630 [99]. In addition, opposite effects on biofilm formation in disruption mutants of *cwp84* in both 630Δ*erm* and R20291 have been observed [36,37], and there is what appears to be a more vital role for type IV pili in R20291 biofilm formation when compared to 630 [60]. Variation in flagellar machinery and associated genes has also been observed between R20291 and 630 strains with respect to arrangement and posttranscriptional modifications as well as phase variation capabilities [55]; however, we can conclude that flagellar genes, and functional flagella, are down-regulated in biofilms [38,52].

The lack of studies to confirm observations made by individual research groups makes it difficult to conclude an absolute pathway for biofilm regulation. Perhaps, importance should be placed on focusing efforts to elucidate the biofilm formation pathway in few select *C*. *difficile* strains before expanding research to increasing numbers of strains with highly variable results. The use of gene expression studies should, in future, allow us to learn more about biofilm regulation in different *C*. *difficile* strains, with the potential for development of improved treatment options for this opportunistic pathogen.
